# Challenges of using modelling evidence in the visceral leishmaniasis elimination programme in India

**DOI:** 10.1371/journal.pgph.0001049

**Published:** 2022-11-29

**Authors:** Natalie J. Dial, Simon L. Croft, Lloyd A. C. Chapman, Fern Terris-Prestholt, Graham F. Medley

**Affiliations:** 1 Department of Global Health and Development, Faculty of Public Health and Policy, London School of Hygiene and Tropical Medicine, London, United Kingdom; 2 Faculty of Infectious and Tropical Diseases, London School of Hygiene and Tropical Medicine, London, United Kingdom; University of Colorado Anschutz Medical Campus: University of Colorado - Anschutz Medical Campus, UNITED STATES

## Abstract

As India comes closer to the elimination of visceral leishmaniasis (VL) as a public health problem, surveillance efforts and elimination targets must be continuously revised and strengthened. Mathematical modelling is a compelling research discipline for informing policy and programme design in its capacity to project incidence across space and time, the likelihood of achieving benchmarks, and the impact of different interventions. To gauge the extent to which modelling informs policy in India, this qualitative analysis explores how and whether policy makers understand, value, and reference recently produced VL modelling research. Sixteen semi-structured interviews were carried out with both users- and producers- of VL modelling research, guided by a knowledge utilisation framework grounded in knowledge translation theory. Participants reported that barriers to knowledge utilisation include 1) scepticism that models accurately reflect transmission dynamics, 2) failure of modellers to apply their analyses to specific programme operations, and 3) lack of accountability in the process of translating knowledge to policy. Political trust and support are needed to translate knowledge into programme activities, and employment of a communication intermediary may be a necessary approach to improve this process.

## Introduction

### Neglected tropical disease elimination

Substantial progress has been made globally over the past decade to interrupt and drive down transmission across the 20 neglected tropical diseases (NTDs) [1]. The World Health Organization (WHO) roadmap for control, elimination, and eradication has now been restructured for 2030 [1], reflecting the fact that reaching and sustaining low-incidence becomes increasingly challenging and less cost-effective as targets are approached. Political, financial, and research momentum must be reinforced to maintain NTD surveillance, substantiate and secure resource allocation, and streamline the evidence to policy process [2, 3]. As the COVID-19 pandemic has additionally postponed fundamental programmes and activities, actionable research is essential for informing the scale, scope, and intensity of intervention strategies required to reach elimination targets and mitigate NTD resurgence [4].

### Visceral leishmaniasis elimination in India

Visceral leishmaniasis (VL), also known as kala-azar, is an NTD with the second highest fatality rate of any parasitic disease, where India accounts for 25% of the global burden [5]. Transmitted by the female phlebotomine sand fly, VL is characterised by fever, anaemia, enlargement of the spleen and liver, and a 95% fatality rate in those untreated [5]. Cases of post-kala-azar dermal leishmaniasis (PKDL), a secondary skin condition of VL, also contribute to transmission [6–9]. VL in India is targeted for elimination as a public health problem (EPHP), which will require long-term surveillance to sustain low-incidence and reduce the risk of both outbreaks and resurgence.

Since its inception in 2005, India’s national Kala-Azar Elimination Programme (KEP) has achieved substantial declines in VL incidence, but an estimated 3,000 cases remain amongst an at-risk population of 150 million people within India’s four VL-endemic north-eastern states [5]. The current WHO target aims to achieve less than 1% case fatality rate due to primary VL and to detect 100% of PKDL cases in India by 2030, partially because of structural difficulties in achieving the 2020 elimination goal of less than one VL case per 10,000 population at the sub-district (or block) level [1, 10]. Early diagnosis, treatment and vector control are pillars of India’s KEP, which are carried out through a combination of active and passive case detection activities alongside indoor residual spraying (IRS) of synthetic pyrethroids [1, 11].

### Modelling to inform elimination strategies

Strategising the scale and scope of elimination activities requires iterative research to identify drivers and dynamics of VL transmission, which is best characterised by rigorous reporting and evaluation of surveillance activities [4]. As ubiquitous and complete disease-specific surveillance is impossible, case detection during pre-elimination must be prudently designed and implemented to minimise uncertainty in reported incidence. However, as disease incidence decreases, so do sample numbers that generate statistical power necessary for programme evaluation [12].

With its capacity to capture and reproduce important features of the real world through simplified structures, mathematical modelling is a compelling research tool for characterising dynamics between those susceptible, exposed, infectious, and recovered from a disease [13, 14]. Modelling has played an integral role in mitigating modes of disease transmission across populations at risk by projecting the dynamics of pathogens, vectors, and hosts, the potential impact of different interventions, and herd immunity thresholds requisite for achieving and sustaining elimination targets [13, 15].

The direct application of modelling to policy, however, is not only context- and disease-specific but also influenced by factors internal and external to the models [16]. For malaria and NTDs eligible for population-level treatment through mass drug administration (MDA), the application of modelling to inform policy and programme design is through evaluation of the intensity and longevity of interventions required to reach elimination targets [15, 17–20]. For VL, modelling must address pathways and outcomes of individualised case finding through ‘innovative and intensified disease management’ (IDM), which complicates how modelling extends to strategising surveillance, interventions, and elimination targets [21–25]. Partnerships and stakeholders within and between modelling and policy realms also affect the translatability of models [26], as does the inclusion or exclusion of economic, social, and political indicators [16].

### Insights from VL modelling in India

Over the past seven years, a marked increase in VL modelling has been sought and accomplished to contribute towards India’s national elimination strategy [27–29]. This body of modelling has spanned various objectives to characterise transmission, the effects of interventions, and the likelihood of reaching elimination, including the improvement of data collection to better inform models themselves. Much of the early modelling on individual-level disease progression, infectivity, and demographic indicators improved knowledge surrounding the natural history of VL [6, 15, 30–32]. More recent transmission models have contributed knowledge that 1) VL and PKDL cases are the main drivers of transmission, 2) the scale and pattern of immunologically naïve individuals in relation to declining incidence is important, and 3) VL incidence can be accurately forecasted up to 4-months ahead over space and time to inform disease management [6, 9, 33–36]. Interventions, particularly those that reduce the time from infection to diagnosis, have also been modelled and compared to the prospect of reaching elimination targets [9, 37–39]. Lastly, the geographical scope and timeline of WHO elimination targets have been modelled to evaluate their feasibility given current strategies and a dynamic landscape of incidence [40].

A literature review was carried out to identify and synthesise key VL modelling studies pertaining to the Indian-Subcontinent (ISC) that were published over the past decade. This review aimed to provide an informative summary of VL modelling literature in the ISC rather than an all-encompassing or exhaustive systematic review. PubMed and Embase were used to search for studies including key terms related to: modelling; visceral leishmaniasis (or Kala Azar); and the Indian-Subcontinent (India, Nepal, and/or Bangladesh). Table 1 outlines all studies identified within this search criteria that have been published since 2011, summarised according to: 1) model type (deterministic or stochastic; static or dynamic; individual or structured; or review); 2) study objectives; 3) model assumptions; and 4) study outcomes and gaps identified.

**Table 1:** Outline of key modelling studies on VL in the ISC by model type, objectives, assumptions, outcomes, and gaps identified.

Model [Ref]	Model type, objectives, & assumptions	Outcomes & gaps identified
Stauch et al. 2011 [34]	Deterministic compartmental model Parameters estimated for transmission and optimising EPHP by comparing treatment-based or vector-based interventions	• Simulation suggested that asymptomatic individuals ineligible for treatment primarily drive transmission • Treatment can reduce prevalence of symptomatic disease, but incidence remains unchanged due to intensity of transmission • Vector-related interventions can reduce prevalence of asymptomatic infections, but must be combined with treatment (especially for active PDKL infections)
Stauch et al. 2014 [41]	Transmission model Investigated transmission thresholds dependent on reduction of sandfly density through IRS, LLIN, or destroying breeding sites	• Simulations suggest that EPHP is possible if sandfly density is reduced by 67% by killing sandflies, or if breeding sites can be reduced by 79% through environmental management • Reduction of vector’s life expectancy is more effective than reduction of breeding site capacity
Chapman et al. 2015 [30]	Multi-state Markov (compartmental) model Using data collected over a 4-year timeframe, key epidemiological parameters were estimated to simulate the duration of asymptomatic infection and the proportion of individuals that develop clinical symptoms	• Probability of progressing to clinical disease is associated with initial seropositivity and seroconversion • Estimated duration of asymptomatic infection was 147 days, and symptomatic infection was 140 days, with 14.7% of asymptomatic individuals developing clinical disease • The extended period of asymptomatic infection is important to transmission, and future interventions must aim to reduce time from onset of symptoms to diagnosis and treatment
Medley et al. 2015 [38]	A) Disease progression and health-seeking behaviour model Modelled progression of disease from symptoms to diagnosis, and progression of health-seeking behaviour B) Transmission model Modelled incidence of infection, delay between infection and onset of symptoms, duration of latent stage, and compared intervention that reduces time to diagnosis to intervention targeting undifferentiated fever stage	• Shortening time from healthcare-seeking to diagnosis is likely to reduce VL incidence dramatically • Maintaining population and health system awareness are key as incidence declines • Use of a moderately sensitive diagnostic applied early in the fever stage could reduce incidence, but high diagnostic specificity is crucial
Das et al. 2016 [6]	Statistical model Examined VL transmission and seroconversion in households with VL, PKDL, and asymptomatic infections	• VL infections are the major reservoir for transmission • PKDL and asymptomatic individuals do not exhibit higher transmission than VL • Early identification and treatment must be priority interventions for reaching EPHP
Hirve et al. 2016 [42]	Review Examining role of asymptomatic infection, VL treatment relapse, and PKDL in VL transmission	• Prevalence of asymptomatic infections was 4–17 times higher than VL infections • Infectiveness of PKDL was 32–53%, meaning infections do contribute to VL transmission • VL relapse is highest in HIV-VL coinfected patients • Modelling outcomes varied, predicting that elimination is unlikely; asymptomatic individuals may account for up to 82% of transmission; VL cases are the main drivers of transmission; or sandfly density and breeding sites must be reduced by 67–79% to reach EPHP
Poche et al. 2016 [43]	Individual-based, stochastic, life-stage-structured model Simulated the impact of interventions on sandfly vector populations based on fipronil-based drugs for cattle	• Simulation indicated fipronil-based drugs are effective in reducing sandfly abundance, depending on timing of administration relative to seasonality of sandfly lifecycle • For cost-effectiveness, model suggested administration between April-August (3 times per year) • Sandfly density could be reduced between 83–97%
Rock et al. 2016 [31]	Review Examined biology and data behind VL models, realistic predictions, effectiveness of interventions, and key issues	• Better documentation and understanding of the natural history of disease, immunity, and stages of infection are key for future modelling • The role of asymptomatic and symptomatic infections to contributing to transmission must be considered alongside parasite-sandfly-vector interaction • Gaps in knowledge around current biological understanding and the impact of diagnosis, treatment, and vector control undermine model capacity, and will be crucial for the next generation of VL modelling
Le Rutte et al. 2016 [33]	Deterministic, age-structured transmission model 3 models with different main reservoirs of infection (asymptomatic, reactivation after initial infection, and PKDL) were developed. Assumptions about the duration of immunity, exposure to sandflies, and optimal versus sub-optimal IRS effectiveness were fitted to the data for different levels of VL endemicity	• Predicted impact of IRS varied substantially between each model • Reaching EPHP targets in the ISC depends on assumptions regarding the main reservoir of infection • The model assuming asymptomatic infections are main drivers of transmission is likely the most realistic based on model fit to data • Reaching EPHP targets is most likely feasible in areas with low- and medium- endemicity and optimal IRS • In highly endemic settings with sub-optimal IRS efficacy, additional interventions (such as ACD) will be required
Le Rutte et al. 2017 [44]	Transmission model: population-based, deterministic, age-structured Compared 3 models assuming different contributors to transmission: symptomatic individuals, asymptomatic individuals, and asymptomatic individuals with vector population dynamics	• EPHP targets are likely to be met in blocks with <10 VL cases per 10,000 population per year using ACD and IRS tools • In blocks with <5 VL cases per 10,000 population per year, increasing the scope and effectiveness of IRS could lead to elimination 1–3 years earlier • All models suggest VL transmission will continue after EPHP targets are met, and surveillance and control must remain until interruption of transmission is achieved
Hollingsworth et al. 2018 [47]	Review Modelling literature is reviewed for 9 NTDs to examine progress towards 2020 goals. For VL, knowledge garnered from statistical, probabilistic, and deterministic age-structured, and transmission models is extrapolated to identify progress and gaps	• Models have contributed to the knowledge base in terms of natural history, duration and progress of disease states, and drivers of transmission • More recent models compare and predict the impact of different interventions on reaching EPHP targets • Policy implications can be generated from the body of modelling during this period, especially for improving access to diagnosis and improving efficacy of IRS • Most models suggest asymptomatic individuals contribute significantly to transmission • The relative infectivity of different disease stages is not well-understood, but must be examined to better define assumptions relating to transmission
Le Rutte et al. 2018 [45]	Transmission models Policy-relevant conclusions are synthesised from recent transmission modelling. A model is generated to predictions VL incidence relating WHO-recommended interventions	• Model suggests that WHO guidelines should be sufficient to reach EPHP targets in areas with medium VL endemicity (up to 5 cases per 10,000 population per year) • Additional interventions may be required in high-incidence regions, but the efficacy of interventions depends on the relative infectiousness at different disease stages • Asymptomatic and PKDL infections pose threats to reaching EPHP • As incidence decreases, the pool of immunologically naïve individuals (and potential for new outbreaks) will increase
Chapman et al. 2018 [32]	Statistical and catalytic models Diagnostic tests were compared to prevalence of infection and age groups to assess trends. Infection prevalence age distribution data was modelled using reverse catalytic model to determine seroconversion rates and immunological responses over different timescales	• The age-independent catalytic model provided the best fit to infection prevalence data • Model suggests infection rates may increase with age • Age patterns in asymptomatic infection vary significantly in the ISC • Infection prevalence increased with age, but acquired immunity may also increase with age • Young children may have lower exposure to sandflies • There is poor standardisation of serological tests, which makes data comparison difficult between studies
Chapman et al. 2018 [39]	Spatiotemporal transmission model Key parameters were determined by fitting a transmission model to geo-located epidemiological data in high VL endemic villages. Bayesian inference framework was developed to account for unknown infection times, missing symptom onset, and recovery times	• Parameter estimates suggest that in high endemic settings, VL risk decreases in relation to distance from a case • At 90m from an infective individual, the risk of developing VL is reduced by half • VL cases contribute significantly more to transmission than asymptomatic individuals • Spatially targeted interventions may be key to reducing VL transmission • Interventions must target a radius >300m around a new case to reduce risk of VL transmission
Barley et al. 2019 [35]	Transmission model Model is developed to quantify risk of VL infection in India and Sudan, relying on prevalence level and control reproductive number	• Value of reproductive number is found to be 60% higher in India than in Sudan • Reproductive number is also found to be most sensitive to the average sandfly biting rate, regardless of regional difference • Treatment rate is found to be most sensitive parameter to VL prevalence • Risk factors associated with vector are identified as more critical to transmission dynamics than factors relating to humans
Chapman et al. 2020 [9]	Spatiotemporal transmission model This study combined xenodiagnoses data with geo-located incidence data to detail the changing roles of VL, PKDL, asymptomatic infection, immunity, and transmission	• Model suggests that while VL cases drive transmission in high incidence areas, the contribution of PKDL increases significantly as VL declines • VL transmission is highly focal: 85% of secondary cases occur <300m from inferred infector • Average time from infector to secondary cases was <4 months for 88% of cases • Time from PKDL infector to secondary VL infection can be up to 2.9 years • Estimated secondary cases per VL and PKDL case varied from 0 to 6, and depended on infector’s duration of symptoms • Prevention of PKDL could reduce VL incidence 25% • Prompt detection and treatment of PKDL is crucial to reaching EPHP
Coffeng et al. 2020 [37]	Transmission model Study aimed to understand how changes in detection delay and population coverage of improved detection impact VL incidence and mortality. Predicted impact of reduced detection delays and increased population coverage	• Improved case detection, either by higher population coverage or by reduced time in detecting symptomatic cases, would cause an initial rise in observed VL incidence followed by a reduction in incidence • Similarly, relaxed detection efforts would lead to an apparent (temporary) one year reduction of VL incidence followed by resurgence • Duration of symptoms is highly associated with detection effort • Effectiveness of case detection activities cannot be based solely on VL incidence • Duration of symptoms in detected cases must be used as an additional indicator of programme performance
Nightingale et al. 2020 [36]	Spatiotemporal statistical model Models were developed for monthly VL case counts at block level to evaluate fit and one-month-ahead predictive power	• Model captured 94% of observed case counts during a 24-month test period • One-, three-, and four-month ahead forecasts demonstrated strong predictive performance • For models informed by routinely collected surveillance data, predictions are sufficiently accurate and precise • Forecasts could be used to guide stock requirements of RDTs and drugs, or target ACD strategies over space and time
NTDMC 2020 [40]	Review To assist the WHO in formulating new guidelines for 2021–2030 NTD roadmap, NTDMC reviewed modelling objectives and outcomes over past decade	• Current EPHP target is feasible for settings with low to medium endemicities • Additional control measures are required for high incidence areas • PKDL must be added to targets, as models simulate PKDL contributes to VL transmission and will pose a barrier to reaching EPHP • VL is highly focal, and hotspots must be prioritised for increased control measures • 2020 target may incentivise countries to not detect and report cases as incidence declines and benchmarks are approached • Future modelling may focus on the risk of recrudescence when interventions are relaxed after EPHP targets are achieved

The 19 published studies included in this table include 11 dynamic transmission models, four reviews, three statistical models, and one individual-based model. A reliance on transmission modelling is common across NTDs and vector-borne diseases, as these parameters help to characterise and address underlying biological mechanisms of disease spread, issues of data availability, and political strategies for control and elimination [15, 46, 47]. Compared to other NTDs, VL required additional time, funding, and momentum to build a modelling base that could inform EPHP strategies, and this decade-long cross-collaboration between disciplines, countries, and modelling institutions directly contributed towards the WHO 2021–2030 Roadmap for NTDs [48, 49].

### Use of modelling in India’s KEP policy

Although these VL models did not include, or necessarily warrant, explicit policy recommendations or responses, they have contributed considerable insight into drivers of transmission, the potential impact of interventions, and whether elimination targets are achievable. The ability of VL modelling to inform surveillance-response activities in India will become increasingly relevant throughout this stage of elimination, especially as cases become more difficult to find and require resources to be allocated efficiently across a heterogenous landscape of incidence [50, 51]. Policy-relevant knowledge garnered from modelling should be examined to support political momentum for reaching EPHP targets against competing disease priorities [52, 53].

Given the volume of VL modelling research now available within the context of India, it is timely to consider the extent to which these individual and collective findings have or could influence the design and implementation of KEP elimination activities. Models are only as robust as the assumptions and data they rely on, and only as influential as their actionability and perceived relevance to policy makers. There is a potential divergence between the goals of researchers and policy makers throughout the development of models for communicable diseases, where the objective of broad epidemiological understanding verses practical management must be addressed and resolved [14].

Modellers recognise the significance of carefully presenting assumptions and comparisons within their evolving evidence base to garner trust and support of policy makers [4]. The NTD Modelling Consortium recently published a guideline of Policy-Relevant Items for Reporting Models in Epidemiology (PRIME) to improve 1) stakeholder engagement, 2) complete model documentation, 3) complete description of data used, 4) communication of uncertainty, and 5) testable model outcomes [54]. Although these PRIME guidelines offer actions that modellers may take, what is crucially missing is the perspective of policy makers and the degree to which policy and its actors must be engaged throughout research formulation, data collection, and interpretation of results.

### Knowledge utilisation to examine modelling in the KEP

This study aims to explore whether, and how, VL modelling research has informed or influenced KEP strategies in India and the ways in which model actionability and perception could be improved. Characterising barriers between information generated from research and what is done in practice requires investigating the ‘know-do’ gap. This study employs the theory of knowledge utilisation to explore the transfer, exchange, diffusion, and implementation of VL modelling research into KEP policy [55–57]. Barriers to the operationalisation of VL modelling are identified from the perspective of those designing, interpreting, and implementing policy related to VL elimination research in India. Exploring policy makers’ perceived value and use of VL modelling will assist researchers in the next iteration of models and politicians in the next iteration of elimination guidelines, which should likewise extend to other NTDs and broader geographical contexts.

## Materials and methods

### Conceptual framework

Knowledge translation (KT) is a dynamic and iterative process that includes the synthesis, dissemination, exchange, and ethically sound application of knowledge to improve health, provide more effective health services, and strengthen healthcare systems [58]. It requires collaboration between researchers, clinicians, policy makers, and communities, and can extend to the design, implementation, or evaluation of public health programmes. There remains an important distinction between information (objective, contextualised facts), knowledge (empirical analyses), and evidence (replicable, hypothesis-driven propositions) in the way scientific results should be packaged and portrayed for policy [59]. VL modelling research in this study is categorised as knowledge, in that it represents individual, and not necessarily comprehensive, studies specific to VL elimination and surveillance programmes in India. However, this body of VL modelling work might also be considered a collection of state-of-the-art frameworks that represent the transmission dynamics and impact of VL interventions according to updated evidence, assumptions, and data.

A review of KT, knowledge-to-action (KTA), and diffusion of innovations theories was carried out to identify a framework that would systematically approach the relationship between knowledge producers and users, as well as examine barriers to the transfer of VL modelling to policy in India [57, 60–62]. Under the umbrella of KT theory, the conceptual framework of knowledge utilisation, developed by Knott and Wildavsky, was chosen for its inclusion of explicit stages that indicate how knowledge or information is received, understood, and referenced by decision makers, and if this knowledge then influences action through the adoption, implementation, and desired results of policy change [63]. Knott and Wildavsky’s knowledge utilisation framework suits the aim of this study by investigating the specific process of both physically and figuratively transferring information from the researcher to the decision maker so that gaps in the process can be identified and analysed as they pertain to the actionability of VL modelling studies in India. These stages of knowledge utilisation, therefore, guided the development of a semi-structured interview questionnaire to explore seven dimensions of the theory’s framework, outlined in Table 2.

**Table 2:** Seven dimensions of the knowledge utilisation framework.

Knowledge Utilisation Dimension	Description
Reception	How relevant knowledge is received
Cognition	How knowledge is read, digested, and understood
Reference	If and how knowledge changed the views, preferences, or understanding of the magnitude or probabilities of the impact
Effort	The process by which knowledge influences action
Adoption	How knowledge is written into policy
Implementation	How knowledge is implemented in the programme
Impact	How and whether a change in policy can influence desired results

#### Data collection

Ethical approval was obtained from the London School of Hygiene and Tropical Medicine in November 2019 (Reference Number 17763), and data collection was conducted from August to October 2020.

#### Participant selection and characteristics

This study targeted international-, national-, and state-level policy makers, programme managers, and researchers involved in designing and implementing VL elimination in India. As VL prevalence is low compared to other infectious diseases, the number of senior policy makers and programme managers was expected to be relatively limited. Purposive sampling drove participant selection for inclusion in the study, which was subsequently expanded by snowball sampling. In-person meetings were not feasible due to the COVID-19 pandemic; therefore, all interviews took place over private and secure video conference calls.

#### Interviews

Twenty-eight policy makers, programme managers, and researchers with knowledge, insight, or experience regarding mathematical modelling for VL in India were initially contacted by email to request participation in the study. Of these, 16 key informants (affiliated with the organisations detailed in Table 3) agreed to participate in an in-depth, semi-structured interview.

**Table 3:** Organisations of key informants included in the study.

Organisation (Abbreviation)
CARE India
Banaras Hindu University (BHU)
Institute of Post-Graduate Medical Education and Research
Médicines Sans Frontières (MSF)
World Health Organization Headquarters (WHO HQ)
World Health Organization South-East Asia Regional Office (WHO SEARO)
Indian Council of Medical Research (ICMR) / Vector Control Research Centre (VCRC) / Rajendra Memorial Research Institute (RMRI)
Drugs for Neglected Diseases Initiative (DND*i*)
Institute of Tropical Medicine (ITM)
PATH
Bill and Melinda Gates Foundation (BMGF)
Bangladesh Ministry of Health and Family Welfare
National Vector Borne Disease Control Programme (NVBDCP), India

Key informants received a digital consent form and information sheet explaining the research project before each interview commenced. The consent form was read aloud by the researcher and key informants were given the opportunity to ask questions and confirm their understanding of anonymity, freedom to decline questions, and ability to end the interview at any time. Formal consent to participate in the study was obtained both verbally (documented by audio recording) and written (digitally) pre- and post-interview. Written consent was also obtained by all 16 participants prior to publication to confirm that excerpts quoted in this research paper were cited using the correct tone, content, and context in alignment with ethical requirements.

Each interview lasted between 35 and 80 minutes. The interview guide was iteratively updated based on key informant responses, especially as new themes emerged surrounding modelling and the policy process in India. Interviews were audio-recorded, transcribed verbatim, and corroborated by notes taken during interviews. Key informants were assigned a unique identifier to ensure anonymity, where interview excerpts and references denote each participant’s overarching affiliation within either ‘research’ (R-) or ‘policy’ (P-) followed by a number. This data is freely available within the Open Science Foundation (OSF) open access public repository (https://osf.io/ujygc/).

### Coding and analysis

Interview transcriptions were analysed using the qualitative software NVIVO 12.6.0 (released in November 2019, QSR International) using a mixed-methods deductive (grounded in knowledge utilisation theory) and inductive (generation of new theories) approach [64]. Codes were organised and compared within each of the seven dimensions of knowledge utilisation (Table 2) and analysed according to converging and diverging views on perceived value and usefulness of VL modelling research to policy makers. Flexibility was retained in analysis to explore shifts in key informant responses regarding linear, relational, and systems-based dynamics of knowledge producers and users, with the aim to highlight and address specific barriers to knowledge utilization [65]. Participant responses are organised in the results section in alignment with the seven dimensions of knowledge utilisation and examined analytically through thematic comparison within the discussion.

## Results

Themes are presented within each of the seven dimensions of knowledge utilisation, supported by direct quotes or in-text paraphrasing, and referenced by each participant’s unique identifier. Although the knowledge utilisation framework suggests a linear path between information reception and its eventual impact via policy, this study considers each dimension impartially without implicit correlation between former and subsequent categories.

### Reception

The majority of participants received information regarding past and ongoing modelling studies at international meetings involving multiple organisations and institutions, in which modelling was one of several foci. Those engaged in modelling itself received new information from either research collaborations or published literature (R1, R4, R6). Each of the 16 participants affirmed that the best timeframe for modellers to formally engage with policy makers is at least once per year, either virtually or in-person. Meetings between researchers and policy makers should focus on discussing and deciding whether modelling results have the potential to update the programme (P4).

Modelling presented alongside other research fields helped to contextualise its purpose and contribution towards VL elimination, and sparked interest in broadening interdisciplinary collaborations between biologists, economists, social scientists, and modellers (R8). One key informant suggested a liaison between researchers and policy makers would aid effective communication:

‘You can’t be a jack of all trades; you can’t be a modeller and also a public health communicator. The team should include public health experts who can be a bridge between modellers and policy makers. In my limited experience, people who are good communicators are not necessarily good modellers, and *vice versa*. We need public health individuals as part of modelling groups who can liaise with policy makers to derive maximum benefit.’ (R8)

### Cognition

Participants expressed varying degrees of understanding modelling research. Within specific study components, the objectives of modelling were most easily digested and comprehended by policy makers. However, a disconnect was reported in objectives prioritised between modellers and policy makers:

‘Sometimes politicians are interested in something different than what we as modellers think is important; sometimes they want us to answer a question that cannot be addressed through modelling. They don’t always understand what the models can and can’t do, and we have to tell them we are limited in what our models can be used for.’ (R6)

Specific equations, parameters, and processes of the models themselves were understood least by Policy makers, as well as by researchers without specific training in modelling (R5 & R8). Policy makers felt comfortable to ask questions and seek clarification directly with researchers to improve their understanding of study outcomes (P7). However, where actionable policy recommendations were not regularly communicated as part of modelling research, or perhaps not warranted, the perceived relevance of modelling to policy makers diminished:

‘Generally, the decision makers think that a model is made because something has to be done as an academic exercise and, based on that, modellers come up with results that only they understand which doesn’t have any practical ground-level reality. My recommendation is for each and every researcher to learn how to describe our processes clearly, comprehensively and intuitively, which is possible. [Modelling] can be explained in a language that is more practical and pragmatic, and it can be interpreted in a way which is actionable giving us estimates or predicted values that are scalable and later on sustainable.’ (R4)

To increase political support and ownership through comprehensive engagement, policy makers should be involved in the modelling process from the beginning stage of identifying knowledge gaps and objectives (R2, R4, R8, P2, and P4).

‘[We should] create a training programme where people from NVBDCP or [the Indian Council of Medical Research] come together for a 2- to 3-week intensive modelling training, and everyone uses their own data, and everything is basic. Like, put in these 5 inputs and produce this; something elementary enough that the programme might see effects of increasing their IRS coverage. I think the programme could feel that modelling is a tool for them to use with their own data and knowhow. But because a training like that hasn’t happened, modelling results are always an outsider coming in with a fully baked model that is always mysterious, and they wonder *how did they get there*? *Especially because I haven’t given them my data*. If it were done more hand-in-hand with the programme from a capacity-building angle, modelling might have more influence on the programme.’ (P5)

Reference

Where different modelling outcomes could influence the magnitude and probability of policy change, key informants expressed resounding interest in case predictions and forecasts at the village level (R1, R2, R3, R5, R6, P3, P4, and P8). Village-level case predictions were perceived as desirable for policy makers to 1) inform a more targeted, coordinated, and actionable response:

‘From the case-level and village-level data, a very valuable thing I would like to know is which are the villages in which I am sure to get a VL case, or at least the highest probability. Some models give me a number of how many blocks might have cases based on looking at the whole of the previous year, but we need to go as micro-level as possible so that we can have a point of action. We need to be able to plan pro-actively and go there well-prepared. If I know that in a block there will be 10 villages with VL cases based on the previous year, then I can pay attention to such villages by intensifying my ACD. That type of research would help for planning.’ (R1)

and 2) improve surveillance for other sources of VL-related transmission:

‘If modelling shows us outbreak predictions for a certain number of cases and we end up seeing in reality that 20–30% of those cases are missing and undiagnosed, then we can plan to improve the surveillance part. The major role I see for modelling predictions is on programme implementation. The foremost thing is for us to find cases at the earliest and not permit a case of VL, PKDL, or HIV-VL so that ultimately, we stop transmission. Once we have that, then diagnostics and treatment are both available so there is not much issue financially for the programme. Surveillance activities need to be improved based on the models.’ (P8)

The next most anticipated modelling outcome was to re-evaluate and substantiate VL elimination targets in India. Key informants referenced one modelling study, in particular, that was ‘used extensively by the programme’ as it ‘gave insight into how realistic it is to reach a target’ (P7):

‘The definition of [EPHP] is based on an annual incidence of VL per lowest administrative unit, the block or subdistrict, below a certain target. One model showed that even with very low transmission, there will always be by coincidence a block or other administrative unit that will in one year exceed that limit. This means the disease is no longer eliminated, which makes it very difficult to evaluate and maintain elimination. If you always have these small pockets that pop up and go away again, that in itself is not a problem if the higher incidence in that specific year is just a coincidence. But it still means you have to send outbreak teams to that area for further investigation and if necessary, do control activities. So that is an important activity for post-elimination control and sustainability, because it will require quite big investments to sustain elimination.’ (P1)

### Effort

Key informants largely viewed researchers, not politicians, as responsible for the provision of information that compels policy change (P2, R4, P4, and P8). When research objectives, methods, results, and limitations were not clearly communicated by researchers, key informants identified that disinterest or distrust arose in policy makers:

‘We need trust from the fraternity of people using modelling results, and that trust will help decision makers to be informed. This is thoroughly missing, and what has happened now is that the difference between a prediction, forecast, and speculation is not clear to anyone. So, programme managers and general people think much of modelling as an educated guess. Every statistic is an educated guess, but that education is informing the randomness that’s taken into account in the systematic results. That structured common sense is required to be explained in a language so that people will stop thinking about modelling as assumption-based speculations.’ (R4)

Modelling assumptions have in part been a result of unknowns in transmission dynamics, for which modelling has mobilised improved data collection. Therefore, on the other hand, some key informants conveyed the value of modelling despite its inherent unknowns:

‘I think despite the unknowns about VL transmission, modelling is still valuable. The data is increasingly solid that asymptomatic carriers are not contributing to transmission but PKDL is. The beauty of a model to me is, you can assume with one symptomatic person there are 10 asymptomatic people that contribute 0% versus 5% to transmission, and you can model different scenarios to understand at what point would we need to intervene. You can have sensitivity analyses that say *we don’t know everything*, *but what if it were this or that and what would the impacts be*? If you knew absolutely every variable then obviously you could model that, but right now what’s appealing is that you can have a set of equations in which you can plug in different variable estimates and get different answers and impacts.’ (P5)

### Adoption

Key informants identified differences between VL and other NTDs in the way modelling is able to explore ongoing intervention strategies and thereby be adopted into policy.

‘For some diseases, modelling is much more direct in that it simulates interventions that you can quickly translate to policy. I think that’s the case to a much lesser extent for VL. Much of the modelling work has been on gaining insight into transmission and the unknowns, like how important is this duration of immunity that we don’t know about? What could be the role of asymptomatics, and if they do play a role then what is their impact? These are higher-level questions and not necessarily informing an ASHA or an IRS spray-man of what to do tomorrow.’ (R6)

To increase the likelihood of adopting modelling outcomes into policy, key informants stated modelling should leverage economic indicators and provide comprehensive projections of programme impact alongside long-term costs (P5 & R8). The importance for models to explore novel strategies, especially during this stage of peri-elimination, was also encouraged:

‘People should not think that innovation and operational research ends now. I think it is important now because when you have a reduced number of cases it becomes more difficult to bring cases further down because of the lack of evidence and knowledge. This is where innovation and research should be supported, and we continue to advocate for this. Otherwise, there is a tendency to think that further research is not required because we’re on the verge of elimination. It took 20 years to reach this space, and if we lose out everything in the next 5 years it will be difficult to restart such activities.’ (R5)

### Implementation

In order for modelling outcomes to be implementable within the KEP, the majority of key informants reported that modellers should improve their understanding of ongoing programme activities, capacity, and infrastructure.

‘Modellers absolutely need to know more about operational activities on the ground. I don’t think modellers personally visit those places. It is very important to look at operations in the field and understand the context, then adjust the data to those realities.’ (P6)

Beyond refining research objectives and contextualizing results for policy, key informants reported that directly observing operational activities was also essential to combat unreliable data and discrepancies in reported coverage of activities (P2 & P4).

‘Once you predict the areas where the elimination programme needs to be, the modellers need to know what is in those areas physically on the ground. If they knew what was on the ground, they would have a better idea of how to come up with a solution. They need to visit the areas where the programme is going on—they need to see the system and what is lacking at the ground level. Being a researcher, sitting at a big institution either in India or in London, it is difficult to compare this to what is going on in the national programme and being delivered.’ (R7)

### Impact

The bureaucracy, policy process, and relationship between WHO and the KEP complicate the observed impact of modelling through policy change.

‘The way in which elimination work is done in India, in particular, is that WHO has a guideline, and you do A, B, C and all the programmes follow A, B, C. Modellers might come in and say *you know*, *if you did A twice*, *skipped B and jumped to C*, *you could see a better impact*. The problem is even if the programme fully believes and trusts this information, they feel their hands are tied because of the way WHO controls policy. So, it’s difficult to see a model have direct programmatic impact.’ (P5)

Key informants acknowledged that even if a policy change were to be achieved, barriers to enforcement may undermine actual impact.

‘The main frustration with WHO is that when you gather the experts and those on top of the knowledge to make reports, recommendations, and research questions, it’s often just a paper that remains on a shelf or in a PDF on a website because those with the power and money decide not to support it.’ (P2)

## Discussion

The value and use of VL modelling research examined in this study was largely perceived as instrumental and conceptual, in that it had identified and addressed unsolved problems relating to VL elimination but had not legitimised concrete policy solutions for the KEP. Participants believed that researchers should improve their communication of models and understanding of operational activities, but that accountability and enforcement between the WHO and KEP remain institutional barriers to policy change. Political trust might be heightened by either engaging policy makers in model interpretation to enhance ownership and understanding of research, or by employing communication intermediaries.

### Assumptions undermine knowledge utilisation

Assumptions were identified as barriers to knowledge utilisation in the way models themselves are designed and presented, but also in how modellers assume programme operations occur and in assumed ownership over knowledge translation in the policy process.

### 1) Model assumptions and uncertainty

As models are an abstraction of reality, their application to programmes and policy can be limited by different assumptions that lead to variability in parameters, especially across space and time [31]. In this, policy makers expressed confusion and distrust surrounding model assumptions and uncertainties, in that they undermined the substance of results by rendering them speculative. Assumptions and uncertainties are inherent elements of modelling and, although they may be mitigated or reduced by optimising model structure, parameters, and fit, the ways in which they are presented and negotiated for end-users hold equal importance [66–68]. From the research perspective, modellers must identify, quantify, and communicate model outcomes in a way that delineates *risk* from *uncertainty* [69]. Uncertainty may exist in the model structure, parameters, or natural variability of temporal and spatial elements, which might be leveraged by identifying how improved data collection and surveillance could minimise uncertainties for future research [46]. From the policy perspective, decision uncertainty might relate to the risk of choosing one alternative course of action over another, for example, in terms of financial or epidemiological risk [70]. There is an opportunity to negotiate the perceived usefulness of models from objective beliefs about their quality towards how they function within broader research, societal, and political environments [68].

Much of the VL modelling accomplished over the past seven years required modellers to first address gaps in the collective understanding of transmission, natural history, and immunity. When less was understood about the drivers and parameters of VL transmission, model assumptions may have dissuaded policy makers from acknowledging their potential usefulness. As VL modelling is increasingly intended for a policy response, its limitations may need to be addressed by encouraging policy makers and researchers to explicitly discuss the meaning and confines of assumptions, how to test them, and what data to collect.

The improvement of trust in VL models may necessitate a new role altogether by employing a communication intermediary, or liaison, between researchers and policy makers. Communication intermediaries must have expertise in the relevant research field to be able to communicate and endorse research, but also have insight into the policy process and actors in the relevant context to identify routes and opportunities for change [71]. Such a role might be stipulated and supported by funders and jointly recruited by researchers and policy makers.

### 2) Modellers’ assumptions of on-the-ground operations

Participants recognised that a strength of modelling is its power to hypothesise a variety of scenarios on a scale to help inform decision making, but many indicated that VL modelling must be improved through its design around and for operationalisation. Successful modelling in other NTD programmes, especially those reliant on MDA, demonstrates that existing processes are easier to refine and improve than roles, resources and activities that are not in place [18–20, 72, 73]. Participants identified that models should not only address ongoing interventions such as ACD and IRS, but that the modellers must also observe these activities on the ground to determine operational gaps, central roles, and scalable activities. Further, participants indicated that without witnessing on-the-ground operations, national programme data lends itself to skewed interpretation and less-realistic analyses. It is important that VL modellers exhibit an understanding of activities, roles, and hierarchies within the KEP and more directly gauge their work towards a policy response.

### 3) Assumptions of ownership over knowledge translation

The process of translating knowledge into policy between researchers, the KEP, and WHO Southeast Asia Regional Office (SEARO) poses challenges. Some participants were discouraged by the prospect of translating modelling into actionable policy due to confusion in assumed power of implementation between the KEP and WHO. Although the KEP ultimately coordinates and enforces activities, they rely on formal technical support and guidance from WHO frameworks that are not always promptly realised into action. Further, WHO frameworks may be too broad and infrequent to align with the timely and policy-relevant results that modelling intends to produce.

An independent assessment was published by researchers, policy makers, and programme managers through WHO in 2020, which suggested modellers publish objectives early and often to improve their usefulness to policy [74]. This study, in contrast, found that the majority of policy makers are currently, and prefer to be, briefed on modelling through in-person partnership meetings. Therefore, it may be beneficial to capitalise on existing platforms and relationships between modellers and policy makers to encourage research actionability in the KEP. Modelling might be more effectively operationalised incrementally through informal routes of policy change by leveraging influence and discussion during partnership meetings.

Eliciting a top-down national- or state-level KEP response may not be feasible or justified from existing VL modelling research, but a bottom-up approach could be useful to inform and evaluate the impact of village- or block-level programme activities. For example, as some participants suggested, a pilot programme to heighten ACD in a village or block where incidence is forecasted to be higher in subsequent months could be feasibly trialled on a small scale. In this, models themselves can be considered a tool to communicate current and prospective policy-relevant research opportunities primed for implementation or scale-up. Knowledge utilisation literature also indicates that research is used in different ways over time by different groups, and the dynamics between knowledge producers, users, and translation can be adjusted during periods of static response [61].

### Co-production and co-interpretation of models

Researchers and policy makers are often viewed separately as ‘two communities’ in public health, and barriers to the translation of research into policy are regularly traced back to the production and communication of scientific knowledge [55]. As producers of knowledge with accountability over analysis and scientific integrity of modelling results, researchers in this study were assumed to hold more influence than policy makers in determining the relevance of models to policy; and although policy makers in the KEP readily support modelling objectives and data collection, they are not consistently included in analysis, interpretation, and therefore application of modelling for policy.

Co-production of research between scientists and policy makers can improve accountability in translating knowledge to policy [75, 76]. Especially as participants identified deficiencies in modellers’ understanding of on-the-ground operations, and researchers likewise identified unrealistic goals or requests from policy makers, co-production of research embeds and is embedded in a more realistic discussion around normative operational practices and analytic techniques [77, 78]. It may be important that all stakeholders aim to reach consensus of modelling results in order to effectively translate research into policy [26].

The fidelity of co-production, however, is often limited by increased time commitment, financial and reputational costs, and power struggles [79]. Co-production should be approached cautiously, as it requires agreement to responsibilities, outputs, and authority that may be unrealistic or even damaging to established partnerships [80]. As an exercise of co-production, participants proposed a modelling workshop giving policy makers the opportunity to formulate, analyse, and interpret models themselves to improve their ownership and understanding of results. This aligns with some findings that co-production is best adopted as an exploratory social practice to shift, but not mandate, conventional dynamics of engagement, credibility, and productivity between researchers and policy makers [81].

### The NTD and policy interface

Barriers to knowledge utilisation identified in this study are reflected in other NTD programmes globally, particularly in contextual governing dynamics and research communication [54, 82]. The process of informing elimination strategies through modelling not only relies on aligning research objectives and national agendas but also on subsequent translation to and observation of WHO guidelines. Other NTD programmes supported by international donors found that silo-ed funding poses additional challenges to implementation and policy influence, which strengthens the case for national-level programme integration and financial ownership [83]. As modelling continues to drive forward innovative and policy-relevant elimination research for NTDs, its application might best be encouraged through informal policy platforms, workshops, and communication intermediaries. Future qualitative studies are necessary to continue identifying context-specific barriers to translating knowledge into policy.

### Limitations

This study aimed to gauge the value and practicality of VL modelling for policy makers in India, which may have resulted in participation, researcher, and sampling biases. Although a strength of qualitative research is its ability to identify complexities and subtleties of issues in depth and detail, it is often subject to issues of rigor, transparency, reliability, and validity compared to quantitative research. Validity, as it relates to the honesty and genuineness of data collected, was addressed by relying on a substantiated and commonly used theoretical framework of knowledge utilisation. Reliability, as it relates to the reproducibility and stability of data, was addressed by including a diversity of actors and presenting coinciding and conflicting responses. Further, the framework of knowledge utilisation allows for reproducibility and transparency of future research conducted in this field.

Although the study was grounded in the theoretical framework of knowledge utilisation, qualitative interviewing is inherently subjective due to the presence and training of the interviewer. The interviewer did not have prior involvement with the modelling work in question, which strengthened the objectivity and neutrality of their perspective. The influence researchers may exert on their findings is a common concern throughout qualitative research and was also addressed by considering reflexivity throughout the process of study design, data collection and analysis.

The number of participants may have resulted in sampling bias, especially as 12 of the 28 individuals invited to participate did not respond. The limited number of policy makers and programme managers working in VL in India was identified prior to conducting interviews but was reduced further by lack of participant availability throughout the COVID-19 pandemic. Sampling bias was addressed by including participants from diverse organisations, countries, and positions in research, programme implementation, and policy, as well as recruiting additional participants through snowball sampling. Participant bias was also addressed by transparently reporting the interviewer’s position, affiliation, and research aims prior to conducting interviews, as well as through framing open-ended and neutral questions.

Participation bias is likely present in that key informants’ perspectives, insight, and experience were influenced by their current job position, organisational affiliation, and field of expertise. Some participants had more technical experience with modelling while others had more technical experience with the policy process and its actors in India. Both perspectives were important to compare and contrast in this study and should provide a more comprehensive understanding of the working relationship between VL researchers, policy makers, and programme managers in India. This qualitative analysis could be strengthened and expanded by further investigating the relationship between modellers and researchers through direct observation during partnership meetings, analysing documents of the KEP and WHO SEARO policy processes in India, and through case studies following the influence of a particular modelling study from inception to reception by policy makers.

## Conclusions

With its capacity to employ diverse variables, illuminate trends and drivers of transmission, and project the likelihood and resources required for reaching elimination, mathematical modelling is an increasingly applicable field for informing VL elimination in India. Through the framework of knowledge utilisation, key informants gauged their reception, understanding, and prioritisation of VL modelling outputs aimed at informing the KEP and identified barriers to action within the policy process. Overarchingly, participants assigned value to the knowledge mathematical modelling has contributed to VL transmission and elimination targets in India, but with reservation surrounding its direct application to ongoing activities. Where objectives, outcomes, and limitations are not effectively communicated, the capacity of modelling to influence policy is undermined. Results of this study suggest there is a gap between modelling and policy action, and that knowledge utilisation may be impeded specifically within the process of interpreting and operationalising modelling findings. The trust and endorsement of modelling might be improved by employing communication intermediaries and engaging policy makers in interpretation of results.
